# Richter转化患者的克隆同源性检测及其分子生物学特征分析

**DOI:** 10.3760/cma.j.issn.0253-2727.2022.10.007

**Published:** 2022-10

**Authors:** 业钦 沙, 睿 姜, 祎 缪, 彤璐 邱, 姝超 秦, 婧妍 邱, 红岭 秘, 微 吴, 纯 乔, 雨洁 吴, 奕 夏, 莉 王, 磊 范, 卫 徐, 建勇 李, 华渊 朱

**Affiliations:** 1 南京医科大学第一附属医院，江苏省人民医院血液科，南京 210029 Department of Hematology, the First Affiliated Hospital of Nanjing Medical University, Jiangsu Province Hospital, Nanjing 210029, China; 2 江苏省人民医院浦口分院血液科，浦口慢淋中心，南京 211800 Pukou CLL Center, Pukou Division of Jiangsu Province Hospital, Nanjing 211800, China

**Keywords:** 白血病，淋巴细胞，慢性, Richter转化, 克隆同源性, Leukemia, lymphocytic, chronic, Richter transformation, Clonality relatedness

## Abstract

**目的:**

探索Richter转化（RT）患者的克隆同源性、临床与分子生物学特征。

**方法:**

回顾性分析南京医科大学第一附属医院血液科（浦口慢淋中心）2019年1月至2021年12月确诊的18例RT患者慢性淋巴细胞白血病/小淋巴细胞淋巴瘤（CLL/SLL）及转化弥漫大B细胞淋巴瘤（DLBCL）的免疫球蛋白重链可变区（IGHV）基因片段使用及IGHV-D-J重排模式，鉴定患者克隆同源性。结合患者初诊及转化时的临床信息及分子检测，分析RT患者的高危因素。

**结果:**

18例RT患者转化时中位年龄56.5（41～75）岁。其中17例转化为DLBCL，1例转化为霍奇金淋巴瘤（HL）。17例RT DLBCL患者中，15例（88.2％）患者DLBCL与CLL/SLL克隆同源；2例（11.8％）患者与CLL/SLL克隆非同源。其中11例患者转化前未接受治疗的CLL/SLL样本与转化后DLBCL样本配对的二代测序（NGS）结果显示突变频率最高的基因均为EGR2、TP53、NOTCH1；但部分患者转化时出现上述基因突变的新获得或丢失，提示存在克隆演变；10例布鲁顿酪氨酸激酶（BTK）抑制剂治疗后转化的患者中4例出现BTK突变。上述突变可能为促进转化的高危因素；此外，TP53、EGR2突变可能为包含新药联合方案治疗RT的不良预后因素。

**结论:**

本中心转化DLBCL患者大多为克隆同源性转化；推荐有条件的中心开展相关检测。转化前未治CLL/SLL与转化后DLBCL组织的突变谱有一定异质性。

## Abstract (en)

**Objective:**

To investigate the clinical, genetic, and clonality related aspects of individuals with Richter transformation（RT）.

**Methods:**

From January 2019 to December 2021, 18 RT patients with diagnoses at the First Affiliated Hospital of Nanjing Medical University（Pukou CLL center）were retrospectively examined. The immunoglobin heavy variable（IGHV）gene usage and IGHV-D-J rearrangement pattern of diagnosed CLL/SLL and transformed diffuse large B-cell lymphoma（DLBCL）were compared to determine the clonality relatedness. To investigate the risk factors of RT, Clinical and laboratory data from patients with newly diagnosed CLL/SLL and transformed DLBCL were gathered.

**Results:**

The median age of RT was 56.5（41–75）years old. 17 patients transformed to DLBCL and 1 transformed to Hodgkin lymphoma（HL）. Of 17 individuals who had DLBCL transformation, 15 had CLL/SLL-related clonality and 2 had unrelated clonality. Next-generation sequencing（NGS）analysis of 11 paired initially diagnosed treatment-naive CLL/SLL and RT DLBCL found that EGR2、TP53 and NOTCH1 were among the most frequently mutated genes both in treatment-naive CLL/SLL and in RT DLBCL. In several cases, specific mutations were gained or lost throughout RT, indicating clonal evolution. Among 10 patients before exposure to BTK inhibitors before RT, four patients acquired BTK mutation. The aforementioned mutations should be considered high-risk variables for transformation; in addition, TP53 and EGR2 mutations could be linked to a poor prognosis following RT in patients receiving a cocktail of new medicines.

**Conclusion:**

Most RT DLBCL patients in our center are clonality related（15/17，88.2％）and we recommend all qualified centers to evaluate clonality relatedness of RT DLBCL patients. There was some variability in the mutational landscape between DLBCL that had undergone a transformation and initially diagnosed, treatment-naive CLL/SLL. The underlying molecular mechanism of RT needs more research.

Richter转化（RT）是一类少见的淋巴造血系统疾病，定义为慢性淋巴细胞白血病/小淋巴细胞淋巴瘤（CLL/SLL）转化为侵袭性淋巴瘤的特殊形式[1]。RT最常见的模式是转化为弥漫大B细胞淋巴瘤（DLBCL），大约占所有转化的90％；另外有少部分患者转化为霍奇金淋巴瘤（HL）、浆母细胞性淋巴瘤以及其他罕见的淋巴瘤形式[2]。RT在CLL/SLL人群中的发生率为2％～10％[3]，新药治疗时代下RT发生率相较于化学免疫治疗时代并未下降[4]，因此仍然是CLL/SLL病程演变及治疗进程中亟需探索的重要问题。

RT被分为转化组织与CLL/SLL克隆相关（RT-DLBCL中约占80％）和与CLL/SLL克隆无关两个主要类别[5]–[6]。既往报道确认同源性转化DLBCL的患者即使在新药时代下中位生存时间仅8个月左右[7]。因此，确定是否为克隆同源性对于判断患者预后及选择治疗方案有非常重要的价值。

本研究基于免疫球蛋白重链可变区（IGHV）基因片段使用和IGHV-D-J重排模式检测，对17例RT DLBCL患者进行克隆同源性鉴定及同型模式判读。我们进一步通过临床回顾性分析及荧光原位杂交（FISH）、染色体核型分析及二代测序（NGS）等分子生物学技术，探索RT的临床及分子生物学高危特征，为患者的预后及治疗选择提供进一步指导。

## 病例与方法

1. 病例：纳入2019年1月至2021年12月在南京医科大学第一附属医院血液科（浦口慢淋中心）就诊的18例RT患者。所有患者均在病情进展时行PET-CT检测，对于代谢摄取最高值/次高代谢摄取位置（最高点不可取）的病灶部位行活检或穿刺后，参照2016年WHO分型诊断标准病理明确诊断RT DLBCL/HL。所有CLL患者初诊时均行外周血流式细胞术免疫表型检测诊断，SLL患者初诊时均行淋巴结及骨髓穿刺活检诊断，诊断标准参照国际CLL（iwCLL）工作组2018指南[8]。

2. 免疫球蛋白基因重排及IGHV突变检测：提取病灶组织（CLL外周血单克隆B细胞≥1×10^7^、病理免疫组化明确为SLL的淋巴结组织或骨髓样本，以及病理明确转化的活检及穿刺组织），应用Invitrogen Dynal DNA纯化试剂盒抽提DNA。使用上海源奇生物医药科技有限公司免疫球蛋白基因重排检测试剂盒（毛细管电泳法）对于免疫球蛋白重链（IGH）基因包括FR1、CDR1、FR2、CDR2、FR3、CDR3、JH在内的全部片段进行扩增，明确IGHV基因片段使用并鉴定克隆性重排。使用功能性重排片段进行直接测序，测序结果应用IMGT/V-QUEST数据库与已知参考序列进行比对分析（http://imgt.cines.fr），将IGHV序列一致性<98％定义为IGHV有突变，一致性≥98％定义为IGHV无突变。

3. NGS检测：使用患者转化前未治疗的CLL/SLL样本（CLL外周血单克隆B细胞≥1×10^7^、病理免疫组化明确为SLL的淋巴结组织或骨髓样本）与转化后DLBCL组织样本分别进行慢性淋巴细胞增生性疾病72分子NGS检测（上海睿昂基因公司检测）。

4. 随访：通过查阅患者纸质及电子病历资料确认患者治疗情况，对患者进行电话随访，随访时间截至2022年2月15日。无进展生存（PFS）时间指患者转化后至第一次发生疾病进展或任何原因死亡的时间。总生存（OS）时间指患者转化后至因任何原因引起死亡的时间。

5. 统计学处理：采用Graphpad Prism 7.0a与R 4.0.2软件进行统计学分析。R包maftools被运用于对17例行NGS检测患者突变谱进行分析。Kaplan-Meier法用于绘制患者生存曲线。Dana-Farber既往报道的537例初诊CLL患者基因突变频率及135例初诊DLBCL患者基因突变频率由cbioportal网站公开获取（https://www.cbioportal.org）。*P*<0.05为差异具有统计学意义。

## 结果

1. RT患者诊断及克隆同源性检测：本研究纳入了2019至2021年在我院确诊的18例CLL/SLL RT患者。其中男10例（55.6％），女8例（44.4％），转化时中位年龄56.5（41～75）岁，转化后中位随访时间13.5（1～38）个月。18例患者均在病情怀疑转化时进行PET-CT检测，其中15例（83.3％）最大标准摄取值（SUVmax）>10，3例（16.7％）SUVmax≤10。对于代谢摄取最高值的病灶部位行穿刺活检后，经病理明确诊断RT，其中17例（94.4％）转化为DLBCL，1例（5.6％）转化为HL。14例转化DLBCL及1例转化HL患者表现为淋巴结部位转化，其中7例RT DLBCL患者骨髓细胞学可见大细胞形态；1例患者表现为骨转化；2例患者骨髓诊断DLBCL转化，形态可见胞体较大淋巴细胞（正常淋巴细胞两倍以上），胞质量丰富，胞核呈多形性，染色质较粗糙，有厚实感，骨髓免疫组化示CD5（+）、CD10（−）、CD20（+++）、PAX5（+++），CD23（少量+）、LEF1（部分+），骨髓流式细胞术前向角散射（FSC）示细胞体积为中等至大细胞，免疫表型部分表达CD5、CD22、CD20、CD79b，符合同源性转化DLBCL免疫表型。为确定RT DLBCL患者克隆同源性，使用RT DLBCL组织进行IGH克隆重排扩增及IGHV基因片段使用检测，与诊断CLL时的IGHV基因片段使用及IGHV-D-J重排模式进行比对，17例中15例（88.2％）两者片段相同，提示DLBCL与既往CLL/SLL克隆同源；另有2例（11.8％）片段使用不同，提示DLBCL为新生病灶，与CLL/SLL克隆无关。17例RT DLBCL患者IGHV基因片段使用中，6例（35.1％）为4-39，其中2例患者归类为同型模式8；另有2例（11.8％）片段使用为3-21，其中1例患者归类为同型模式64B。其中1例患者初诊CLL样本及DLBCL转化样本中均检出4-39及5-51两个IGH克隆。2例非同源转化患者初诊时均为SLL，其SLL IGHV基因片段使用分别为1-69及6-1，转化后DLBCL组织IGHV基因片段使用均为3-53（图1）。

**图1:**
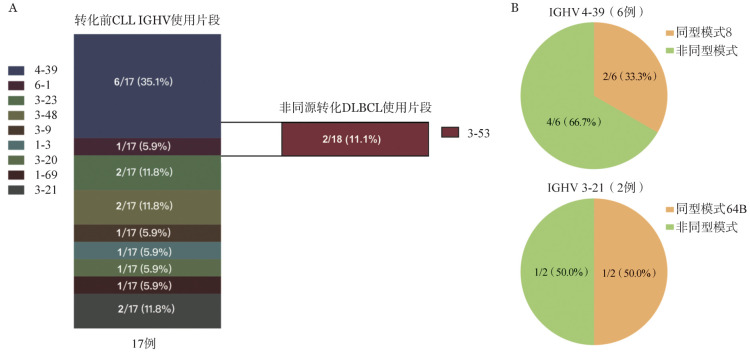
17例RT DLBCL患者CLL/SLL及DLBCL组织IGHV片段使用（A）及IGHV4-39、IGHV3-21同型模式分析（B） RT：Richter转化；DLBCL：弥漫大B细胞淋巴瘤；CLL/SLL：慢性淋巴细胞白血病/小淋巴细胞淋巴瘤；IGHV：免疫球蛋白重链可变区

2. RT患者的临床及病理特征：在本研究纳入的18例RT患者中，初诊未治时临床表现为CLL的患者7例（38.9％），临床表现为SLL的患者11例（61.1％）。其中，17例（94.4％）患者为IGHV无突变，仅1例（5.6％）患者为IGHV有突变。17例转化DLBCL患者中，中高危及高危（DLBCL IPI评分≥3）患者10例（58.8％），11例（64.7％）患者乳酸脱氢酶升高，6例（35.3％）患者为大包块。在15例淋巴结组织穿刺活检病理免疫组化明确转化为DLBCL的患者中，1例（6.7％）为生发中心起源型（GCB），14例（93.3％）为非生发中心起源型（non-GCB）。18例患者中，2例（11.1％）既往单纯接受靶向治疗，2例（11.1％）单纯接受化学免疫治疗，8例（44.5％）接受靶向及化学免疫治疗，另外有6例（33.3％）在初诊未治状态即发生组织学转化（表1）。

**表1:** 18例Richter转化患者临床特征分析［例数/分析例数（％）］

特征	数值
年龄	
≥65岁	3/18（16.7）
<65岁	15/18（83.3）
性别	
男	10/18（55.6）
女	8/18（44.4）
初诊时疾病状态	
CLL	7/18（38.9）
SLL	11/18（61.1）
初诊时疾病分期	
CLL Rai 0~Ⅱ Binet A-B	4/7（57.1）
CLL Rai Ⅲ~Ⅳ Binet C	3/7（42.9）
SLL ⅣA	9/11（81.8）
SLL ⅣB	2/11（18.2）
转化疾病类型	
DLBCL	17/18（94.4）
cHL	1/18（5.6）
DLBCL同源性检测	
同源	15/17（88.2）
非同源	2/17（11.8）
IGHV突变状态	
无突变	17/18（94.4）
有突变	1/18（5.6）
转化时PET-CT	
SUVmax >10	15/18（83.3）
SUVmax ≤10	3/18（16.7）
转化DLBCL后IPI评分	
0～2	7/17（41.2）
3～5	10/17（58.8）
转化DLBCL后LDH>250 U/L	11/17（64.7）
转化DLBCL后大包块（>5 cm）	6/17（35.3）
DLBCL细胞起源	
生发中心起源	1/15（6.7）
非生发中心起源	14/15（93.3）
既往暴露治疗	
单纯靶向治疗	2/18（11.1）
单纯化学免疫治疗	2/18（11.1）
靶向与化学免疫治疗	8/18（44.5）
未治疗	6/18（33.3）

注：CLL：慢性淋巴细胞白血病；SLL：小淋巴细胞淋巴瘤；DLBCL：弥漫大B细胞淋巴瘤；cHL：经典型霍奇金淋巴瘤；IGHV：免疫球蛋白重链可变区；SUVmax：最大标准摄取值；IPI：淋巴瘤国际预后评分；LDH：乳酸脱氢酶

3. RT患者的分子生物学特征研究：对本研究纳入的17例RT DLBCL中11例患者的初诊CLL/SLL未治疗样本及其转化后RT DLBCL组织样本匹配的NGS结果进行分析。转化前初诊未治样本中中位肿瘤突变负荷为2个，突变频率最高的基因依次为EGR2（6/11, 54.5％）、TP53（3/11, 27.3％）、ATM（3/11, 27.3％）、NOTCH1（2/11, 18.2％）；转化后RT DLBCL组织中中位肿瘤突变负荷为3个，突变频率最高仍为EGR2（5/11, 45.5％）、TP53（3/11, 27.3％）、ATM（3/11, 27.3％）、NOTCH1（3/11, 27.3％）、BTK（4/11，36.4％），分析可获得的16例RT DLBCL组织NGS结果证实有相似的突变谱。值得注意的是，例2中初诊SLL样本中检出TP53剪切位置突变，而RT 后组织中未测得；而NOTCH1突变仅在转化后样本中新检出。非同源转化的例12中转化前样本无TP53、MYC错义突变，而在转化后DLBCL组织样本中检出。在例4转化前样本检出EGR2突变，而转化后未检出；此外，队列中共有10例患者BTK抑制剂治疗后转化，其中4例患者转化后DLBCL组织检出BTK突变（4/10，40％），另有1例患者转化后检出PLCγ2突变（1/10，10％）。具体突变类型及其与克隆同源性的关系见图2。发生RT的患者转化前CLL突变谱与既往报道的初诊CLL人群突变谱，转化后 DLBCL突变谱和原发DLBCL突变谱均有很大差异。16例转化后DLBCL突变谱中，检出突变频率最高的基因依次为TP53突变（6/16，37.5％），EGR2突变（6/16，37.5％），NOTCH1突变（5/16，31.3％）。进一步分析RT DLBCL分子突变情况与预后的相关性发现，EGR2突变患者转化后中位PFS时间为3个月，无突变患者转化后中位PFS时间为21个月，差异有统计学意义（*P*＝0.016）；TP53突变与无突变患者的中位OS时间均未达到（*P*＝0.075）。NOTCH1突变、BTK突变、ATM突变与本中心队列患者预后均未发现显著相关性。RT DLBCL分子突变特征与转化后患者预后的相关性，可能需要更长时间随访及更大的样本量进行进一步探索（图3）。

**图2:**
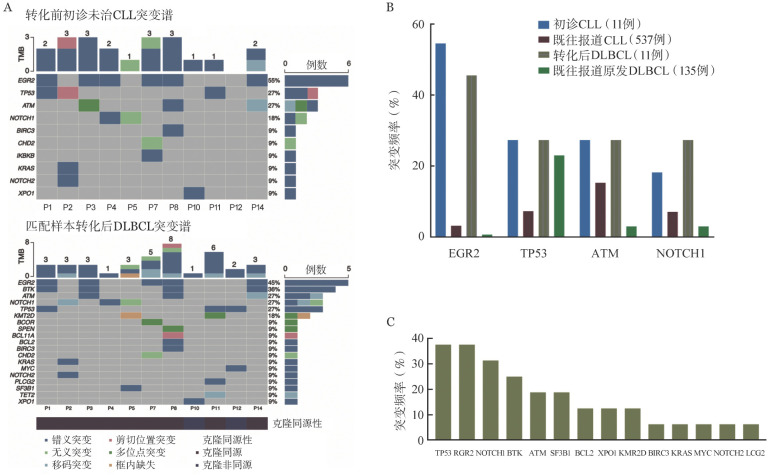
Richter转化（RT）弥漫大B细胞淋巴瘤（DLBCL）患者突变类型及其与克隆同源性的关系 A：RT患者转化前初诊未治慢性淋巴细胞白血病（CLL）及匹配的转化后DLBCL突变谱呈现；B：RT患者转化前CLL突变谱与既往报道的初诊CLL人群及RT患者转化后 DLBCL突变谱与原发DLBCL突变谱比较；C：16例RT患者转化后DLBCL样本分子突变频率

**图3:**
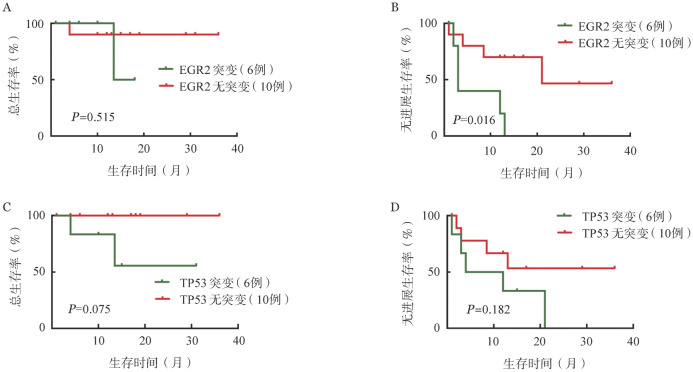
EGR2突变、TP53突变对Richter转化（RT）弥漫大B细胞淋巴瘤（DLBCL）患者预后的影响 A：EGR2突变对总生存的影响；B：EGR2突变对无进展生存的影响；C：TP53突变对总生存的影响；D：TP53突变对无进展生存的影响

## 讨论

RT因其发病率低、预后极差、起病隐匿、分子机制不明成为CLL/SLL治疗领域的难题之一。目前RT患者转化后仍无有效治疗方案，新药的使用在各个临床试验也并未降低RT的发生率[9]–[12]。因此，探讨RT临床及分子生物学特征，对及时发现患者RT证据、精准判断RT患者预后、选择有效的治疗模式具有重要价值。

RT通常以淋巴结及骨髓起病，起病较为隐匿，因此推荐患者在CLL/SLL疾病进展时进行PET-CT检测，在PET指导下对于病灶最高代谢摄取位置进行病理活检或穿刺，有助于RT的及时检出。Michallet等[13]的研究提出SUVmax>10可以作为RT的重要标志，其敏感性（91％）与特异性（95％）均较高。本中心既往研究回顾性分析14例RT患者的PET-CT结果提示，SUVmax的最佳临界值为6.4，此时敏感性为92.9％，特异性为84.2％[14]。本研究纳入的18例RT患者中，15例患者SUVmax>10，提示PET-CT对于RT患者检出具有重要意义。经穿刺活检确诊DLBCL后，建议有条件的中心开展RT DLBCL同源性鉴定。鉴定RT DLBCL患者克隆同源性的最经典方式为IGHV基因片段使用及克隆性IGH重排模式鉴定，患者CLL/SLL组织样本与病理确诊的转化DLBCL组织样本具有相同的IGHV基因片段使用及IGHV-D-J重排模式被认为是同源性RT[3],[6]。单纯依据免疫球蛋白轻链限制性判断RT转化同源性被认为是证据不充分的[15]。此外是否存在新的生物学标志物可以辅助克隆同源性鉴定仍有待于探索，其中PD-1被认为是一个鉴定克隆同源性的标志物，克隆同源性的转化DLBCL组织中高表达PD-1，而克隆非同源的DLBCL组织未见PD-1高表达；克隆同源性的转化DLBCL组织部分表达CD5、CD23，而克隆非同源的DLBCL组织CD5、CD23表达常为阴性[16]–[17]。RT DLBCL转化组织CD5、CD23及PD-1表达在鉴别克隆同源性中的价值需要进一步的研究进行验证。目前基于RT患者CLL样本及转化DLBCL组织样本IGHV基因使用及IGHV-D-J重排模式的检测对于RT DLBCL克隆同源性的鉴定仍具有不可替代的重要价值。

RT的高危分子生物学因素在既往研究中广泛探讨。Rossi、Chigrinova及Fabbri等多项研究[5],[18]–[19]指出，在化学免疫治疗时代，RT DLBCL突变谱与原发DLBCL具有很大差异；60％的RT患者存在TP53基因缺失或突变，并且在克隆同源性的患者中更为常见。30％的RT患者存在NOTCH1基因获得性功能突变，并经常与12号染色体三体伴发。IGHV4-39片段使用以及其中同型模式8（IGHV4-39/IGHD6-13）的患者被认为是发生RT高风险人群，5年内发生RT风险高达68.7％[20]–[21]。本研究纳入的17例RT DLBCL患者中，6例患者为IGHV4-39片段使用，显著高于其他IGHV基因使用片段；其中2例（33.3％）患者经鉴定为同型模式8。对于本研究中完善NGS的11例RT患者转化前初诊未治CLL/SLL样本与转化后DLBCL组织回顾性分析发现，TP53变异与NOTCH1突变频率较高，与既往西方人群研究报道结论一致；同时发现EGR2突变频率在本中心RT患者中较高（6/16, 37.5％），且与患者转化后PFS显著相关（*P*＝0.017），提示RT患者预后不良。尽管Young等[22]的既往研究表明EGR2突变是CLL/SLL患者的不良预后因素，然而其在RT中的预后价值报道尚属首次。关于新药时代下RT DLBCL患者分子生物学特征仍然鲜有文献报道，Kadri等[23]首次报道了6例伊布替尼治疗后进展的RT患者的分子生物学特征，其中4例患者包含BTK位点突变且与TP53突变伴发。本研究中，有10例患者既往使用BTK抑制剂治疗后进展，其中4例患者出现BTK突变，另有1例患者出现下游PLCγ2突变。BTK及PLCγ2突变致使CLL患者对于BTK抑制剂产生获得性耐药[24]。使用伊布替尼治疗后进展为RT的患者中，BTK及PLCγ2突变的比例低于无组织学转化的伊布替尼治疗后进展CLL患者人群[3]，提示伊布替尼治疗下发生DLBCL组织学转化可能有着更多独立于BTK及PLCγ2突变介导耐药的分子生物学机制，有待于更多的机制研究探讨。此外，新药时代下RT DLBCL患者转化后的治疗模式及预后近年来也得到广泛探讨，包括PD-1单抗及CAR-T等免疫治疗，小分子抑制剂联合传统化学免疫治疗可能使患者生存获益。本中心18例患者中，8例既往未暴露于维奈克拉的患者转化后一线接受了VR-DA-EPOCH方案治疗，该方案具有较高的反应率和完全缓解率；但此类患者仍需要桥接包括CAR-T、异基因造血干细胞移植等在内的巩固治疗，以提高远期生存；其余患者也尝试接受了其他新药联合方案。18例患者中位随访13.5个月，中位PFS时间13个月，中位OS时间未达到，提示新药联合方案可能使转化后患者预后获益；然而，生存分析仍提示在包含新药的联合治疗下，RT DLBCL组织中检出EGR2、TP53突变患者预后不佳，提示新药仍无法完全克服不良分子生物学因素，需要结合疾病的生物学特征进一步设计临床试验探索RT患者的个体化治疗选择。

综上所述，本研究基于国内RT DLBCL患者队列进行了克隆同源性检测方法的鉴定，推荐有条件的中心开展相关检测。11例配对的RT患者转化前初诊未治CLL/SLL突变谱与转化DLBCL组织突变谱有一定异质性，RT的具体机制及克隆演化模式仍有待进一步探索。

## References

[1] Swerdlow SH, Campo E, Pileri SA (2016). The 2016 revision of the World Health Organization classification of lymphoid neoplasms[J]. Blood.

[2] Bockorny B, Codreanu I, Dasanu CA (2012). Hodgkin lymphoma as Richter transformation in chronic lymphocytic leukaemia: a retrospective analysis of world literature[J]. Br J Haematol.

[3] Petrackova A, Turcsanyi P, Papajik T (2021). Revisiting Richter transformation in the era of novel CLL agents[J]. Blood Rev.

[4] Ding W (2018). Richter transformation in the era of novel agents[J]. Hematology Am Soc Hematol Educ Program.

[5] Rossi D, Spina V, Deambrogi C (2011). The genetics of Richter syndrome reveals disease heterogeneity and predicts survival after transformation[J]. Blood.

[6] Rossi D, Gaidano G (2016). Richter syndrome: pathogenesis and management[J]. Semin Oncol.

[7] Eyre TA, Schuh A (2017). An update for Richter syndrome - new directions and developments[J]. Br J Haematol.

[8] Hallek M, Cheson BD, Catovsky D (2018). iwCLL guidelines for diagnosis, indications for treatment, response assessment, and supportive management of CLL[J]. Blood.

[9] Maddocks KJ, Ruppert AS, Lozanski G (2015). Etiology of Ibrutinib Therapy Discontinuation and Outcomes in Patients With Chronic Lymphocytic Leukemia[J]. JAMA Oncol.

[10] Byrd JC, Furman RR, Coutre SE (2015). Three-year follow-up of treatment-naïve and previously treated patients with CLL and SLL receiving single-agent ibrutinib[J]. Blood.

[11] Stilgenbauer S, Eichhorst B, Schetelig J (2016). Venetoclax in relapsed or refractory chronic lymphocytic leukaemia with 17p deletion: a multicentre, open-label, phase 2 study[J]. Lancet Oncol.

[12] Jones JA, Mato AR, Wierda WG (2018). Venetoclax for chronic lymphocytic leukaemia progressing after ibrutinib: an interim analysis of a multicentre, open-label, phase 2 trial[J]. Lancet Oncol.

[13] Michallet AS, Sesques P, Rabe KG (2016). An 18F-FDG-PET maximum standardized uptake value > 10 represents a novel valid marker for discerning Richter's Syndrome[J]. Leuk Lymphoma.

[14] 郑 鑫琪, 朱 华渊, 丁 重阳 (2020). PET/CT在Richter综合征中的诊断价值[J]. 中华血液学杂志.

[15] Khan M, Siddiqi R, Thompson PA (2018). Approach to Richter transformation of chronic lymphocytic leukemia in the era of novel therapies[J]. Ann Hematol.

[16] Behdad A, Griffin B, Chen YH (2019). PD-1 is highly expressed by neoplastic B-cells in Richter transformation[J]. Br J Haematol.

[17] He R, Ding W, Viswanatha DS (2018). PD-1 Expression in Chronic Lymphocytic Leukemia/Small Lymphocytic Lymphoma (CLL/SLL) and Large B-cell Richter Transformation (DLBCL-RT): A Characteristic Feature of DLBCL-RT and Potential Surrogate Marker for Clonal Relatedness[J]. Am J Surg Pathol.

[18] Chigrinova E, Rinaldi A, Kwee I (2013). Two main genetic pathways lead to the transformation of chronic lymphocytic leukemia to Richter syndrome[J]. Blood.

[19] Fabbri G, Khiabanian H, Holmes AB (2013). Genetic lesions associated with chronic lymphocytic leukemia transformation to Richter syndrome[J]. J Exp Med.

[20] Rossi D, Spina V, Cerri M (2009). Stereotyped B-cell receptor is an independent risk factor of chronic lymphocytic leukemia transformation to Richter syndrome[J]. Clin Cancer Res.

[21] Rossi D, Spina V, Bomben R (2013). Association between molecular lesions and specific B-cell receptor subsets in chronic lymphocytic leukemia[J]. Blood.

[22] Young E, Noerenberg D, Mansouri L (2017). EGR2 mutations define a new clinically aggressive subgroup of chronic lymphocytic leukemia[J]. Leukemia.

[23] Kadri S, Lee J, Fitzpatrick C (2017). Clonal evolution underlying leukemia progression and Richter transformation in patients with ibrutinib-relapsed CLL[J]. Blood Adv.

[24] Woyach JA, Furman RR, Liu TM (2014). Resistance mechanisms for the Bruton's tyrosine kinase inhibitor ibrutinib[J]. N Engl J Med.

